# Engineering of TM1459 from *Thermotoga maritima* for Increased Oxidative Alkene Cleavage Activity

**DOI:** 10.3389/fmicb.2016.01511

**Published:** 2016-09-22

**Authors:** Matthias Fink, Sarah Trunk, Mélanie Hall, Helmut Schwab, Kerstin Steiner

**Affiliations:** ^1^Austrian Centre of Industrial BiotechnologyGraz, Austria; ^2^Department of Chemistry, University of GrazGraz, Austria; ^3^Institute of Molecular Biotechnology, Graz University of TechnologyGraz, Austria

**Keywords:** alkene cleavage, enzyme engineering, styrene, cupin, TM1459

## Abstract

Oxidative cleavage of alkenes is a widely employed process allowing oxyfunctionalization to corresponding carbonyl compounds. Recently, a novel biocatalytic oxidative alkene cleavage activity on styrene derivatives was identified in TM1459 from *Thermotoga maritima*. In this work we engineered the enzyme by site-saturation mutagenesis of active site amino acids to increase its activity and to broaden its substrate scope. A high-throughput assay for the detection of the ketone products was successfully developed. Several variants with up to twofold improved conversion level of styrene derivatives were successfully identified. Especially, changes in or removal of the C-terminus of TM1459 increased the activity most significantly. These best variants also displayed a slightly enlarged substrate scope.

## Introduction

The carbonyl functionality is widespread in nature and can be found in a broad range of compounds, including metabolites or hormones, in form of aldehyde and ketone. Small aldehydes and ketones, due to their volatility and resulting olfactive property, are largely employed in the fragrance and flavor industry, a billion dollar business (Fahlbusch et al., 2003; Carroll et al., 2016). In addition, aldehydes and ketones are important precursors for pharmaceuticals on industrial scale and, as a result of the polarity and reactivity of the carbonyl group, can easily be transformed to alcohols, amines, cyanohydrins, nitroalcohols, carboxylic acids and esters, or alkanes (Goldberg et al., 2007; Fuchs et al., 2015; Steiner et al., 2015). Aldehydes and ketones are accessible by many synthetic routes, one important tool being the oxidative cleavage of alkenes. Chemical approaches such as ozonolysis or metal-based methods display several drawbacks, including explosive character of the intermediates generated or low yield and poor chemoselectivity (Criegee, 1975; Spannring et al., 2014). Several enzymes are known to catalyze oxidative alkene cleavage reactions to different extent – in many cases as minor (undesired) side reaction, such as heme-dependent enzymes with oxygen (Bugg, 2011; Efimov et al., 2011) or hydrogen peroxide as oxidant (Ortiz de Montellano et al., 1987; Tuynman et al., 2000; Bougioukou and Smonou, 2002) or iron and non-iron metal-dependent enzymes (Leuenberger et al., 2001; Marasco and Schmidt-Dannert, 2008). They belong to different enzyme classes including peroxidases, mono- and dioxygenases or laccases and display different protein structures, and accordingly the reaction mechanisms (if known) differ (Rajagopalan et al., 2013a). One of the most efficient examples, where alkene cleavage is the main reaction, is a Mn(III)-dependent proteinase A like enzyme from *Trametes hirsuta* FCC 047 which cleaves C = C double bonds adjacent to phenyl groups (Mang et al., 2006, 2007; Lara et al., 2008; Rajagopalan et al., 2013b).

Recently, biocatalytic oxidative alkene cleavage activity was also identified in TM1459 from *Thermotoga maritima*, an up to then uncharacterized metalloprotein with cupin fold, which preferentially binds manganese (Hajnal et al., 2015). TM1459 was found active in the oxidative cleavage of styrene derivatives, working under mild conditions and using organic hydroperoxide and molecular oxygen as oxidant pair. As *Thermotoga maritima* is a hyperthermophilic organism, TM1459 is still active at 70°C, however, as the overall recovery of substrate and product was low at elevated temperatures, and organic hydroperoxide tends to rapidly degrade, the conversions were routinely performed at 30°C. Ethylacetate was used as co-solvent to allow the use of increased substrate concentration. So far, the substrate scope of the wild type enzyme is limited and conversion levels are only moderate. As TM1459 can be produced in exceptionally high yield (more than 50% of total soluble protein) in *Escherichia coli* and the enzyme is highly temperature and solvent stable, we aimed to improve the activity and substrate scope of TM1459 by enzyme engineering, making it a potent biocatalyst for the synthesis of valuable compounds.

## Materials and Methods

### General

All chemicals were purchased from Sigma-Aldrich or Carl Roth GmbH, if not stated otherwise. *p-*Chloro-acetophenone (97%) and *p-*Chloro-α-methylstyrene (95%) were obtained from Lancaster (Alfa Aesar, Ward Hill, MA, USA). Materials for molecular biology were obtained from Thermo Fisher Scientific, if not specifically mentioned. *E. coli* TOP10F’ was used for plasmid propagation. For expression of the proteins, *E. coli* BL21 (DE3) Gold was used. The bacterial strains were obtained from Life Technologies (Carlsbad, CA, USA). All primers and gBlocks were obtained from Integrated DNA Technologies, Inc. (Coralville, IA, USA). Lysogeny broth agar plates (LB-Agar (Lennox) were supplemented with 40 μg/mL of kanamycin (LB-Kan). For cultivation in liquid media, LB medium (Lennox) with 40 μg/mL of kanamycin was used. DNA concentrations were measured on a NanoDrop 2000 Spectrophotometer from Thermo Fisher Scientific Inc. Protein concentrations were routinely determined with the Bio-Rad Protein Assay (Bio-Rad, Hercules, CA, USA). For sodium dodecyl sulfate polyacrylamide gel electrophoresis (SDS-PAGE), NuPAGE^®^ 4–12% Bis-Tris Gels, 1.0 mm (Life Technologies, Carlsbad, CA, USA) were used with NuPAGE MES SDS Running Buffer.

### Cloning and Mutagenesis

Site-saturation libraries of active site amino acids (Arg39, Phe41, Ile49, Trp56, Ile60, Phe94, Phe104, Cys106, and Ile108) were generated using primers with NNK codons. The plasmid pET26b(+)-TM1459 (Hajnal et al., 2015) was used as template. For subsequent double and triple mutants, site-directed mutagenesis was performed using plasmids with TM1459 carrying one or two of the desired mutations as templates. All primer sequences are listed in Supplementary Table 1. Mutations were introduced by overlap-extension PCR. The genes coding for the C-terminally truncated variants were generated using a single PCR run. The genes for the three multi-muteins without C-terminal truncation were ordered as gBlocks, which were amplified by PCR. The purified PCR products were re-ligated into the expression vector pET26b(+), which was linearized by NdeI/HindIII HF (New England Biolabs, Inc., NEB, Ipswich, MA, USA). For the ligation with a T4 ligase (NEB) a vector/insert ratio of 1:3 was chosen and the reaction mixture was incubated at 22°C for 90 min, inactivated for 10 min at 64°C and finally desalted. Electrocompetent *E. coli* TOP10F’ cells were transformed with the desalted product and after regeneration the cells were plated on LB-Kan agar. After confirmation of the presence of the insert by colony PCR, plasmids were isolated using the GeneJET Plasmid-Miniprep Kit. Isolated plasmids were sequenced (Microsynth AG Vienna) and used to transform the expression strain *E. coli* BL21 (DE3) Gold. In case of site-saturation libraries, after 1 h kanamycin (40 μg/mL) was added to the cell suspension and it was incubated at 37°C for another 3 h. These cells were then used to inoculate overnight cultures (ONCs) of 5 mL LB-Kan medium. The ONCs were then used to isolate a mix of plasmids with all possible mutations. *E. coli* BL21 (DE3) Gold cells were transformed with the mixed plasmid preparation. Five plasmids per library were sequenced for evaluation of the library quality. 176 colonies per library were cultivated in deepwell plates.

### Deepwell Plate Fermentation

For ONCs 750 μL LB-Kan medium per well were inoculated with single colonies from agar plates using sterile toothpicks and incubated at 300 rpm and 37°C in an orbital shaker. As controls *E. coli* BL21 (DE3) Gold cells harboring pET26b(+)-TM1459 and pET26b(+) without insert were used in quadruplicate. For the main culture, 25 μL of ONC per well were transferred to 750 μL LB-Kan medium supplemented with 100 μM MnCl_2_. The main culture was again incubated at 300 rpm and 37°C. After 6 h protein expression was induced by adding isopropyl-β-D-thiogalactopyranoside (IPTG, final concentration 0.1 mM). The incubation was continued at 25°C and 300 rpm for another 17 h, before the cells were harvested by centrifugation at 3,200 × *g* for 15 min. The supernatant was decanted and the deepwell plates containing the pellets were frozen at -20°C. Moreover, the cultures in the ONC plates were mixed with 400 μL of sterile 50% glycerol per well and frozen at -20°C for cryopreservation. These plates were later used as source for inoculating the rescreening plates.

### High-Throughput Assay

Three hundred μL/per well of lysis buffer (0.28 mg/mL lysozyme from chicken egg white (∼70,000 U/mg), 4 U/mL Benzonase^®^ Nuclease (purity > 90%, 250 U/μL, Merck KGaA), 0.2% Triton X-100 in 50 mM NaPi, pH 8) were added directly onto the frozen pellets and the plates were vortexed until the pellets were resuspended. The plates were shaken at 1,000 rpm for 1 h at room temperature and were subsequently centrifuged for 15 min at 3,200 × *g*. 130 μL of lysate per well were transferred to microtiter plates. 50 μL of substrate mix per well were added to the lysate. The substrate mix consisted of 46% ethanol, 36 mM α-methylstyrene (final concentration in reaction 10 mM), 100 mM *tert*-butyl hydroperoxide (final concentration in reaction 28 mM) and 0.2% Triton X-100 in 50 mM NaPi, pH 8 buffer. The plates were sealed with adhesive film and incubated for 2 h at 37°C. After incubation, 50 μL of detection mix per well were added. For the preparation of the detection mix, vanillin (350 mg/mL) was dissolved in ethanol. One part of the vanillin solution was then mixed with nine parts of 2.2 M NaOH and used immediately. The plates were sealed again and incubated at 37°C to promote the formation of the color complex. After 30 min at 37°C, the absorption was measured at 450 or 442 nm in a plate reader (FLUOstar Omega plate reader (BMG Labtech GmbH, Ortenberg, Germany) or EON plate reader (BioTek Instruments GmbH, Bad Friedrichshall, Germany). The expression of the enzyme variants was verified by analyzing lysate samples from the deepwell plates by SDS-PAGE.

### Shaking Flask Fermentation and Protein Purification

Overnight cultures (50 mL LB-Kan medium in 100 mL shaking flasks) were inoculated with *E. coli* BL21 (DE3) Gold carrying pET-26b(+) constructs and then incubated at 37°C over night in an orbital shaker at 120 rpm. For the main cultures, baﬄed 1 L shaking flasks containing 400 mL of LB-Kan medium supplemented with 100 μM MnCl_2_ were inoculated to an OD_600_ of 0.1 and incubated at 37°C in an orbital shaker at 120 rpm. When an OD_600_ of 0.8 was reached, protein expression was induced by addition of IPTG to a final concentration of 0.1 mM. After induction, the temperature was reduced to 25°C for 20 h. The cells were finally harvested by centrifugation for 15 min at 4°C and 5,000 × *g* and stored at -20°C until further use.

The weighed pellets were resuspended in cold 50 mM NaPi, pH 7, and disrupted by sonication with a Branson Sonifier S-250 for 6 min at 80% duty cycle and 70% output control. Sonication was carried out on ice. The cell lysates were centrifuged for 1 h at 50,000 × *g*. Protein expression was confirmed by SDS-PAGE.

Heat purification of cell-free lysate was performed in 2 mL microreaction tubes. The tubes were incubated for 10 min at 75°C and 600 rpm in a thermomixer (comfort, Eppendorf, Hamburg, Germany) and then centrifuged for 5 min at 20,000 × *g* to clear the lysate of precipitated protein. The protein concentration was determined and the purity of the samples was analyzed by SDS-PAGE. The samples were diluted to 1.0 mg/mL for microtiter plate assays and to 1.2 mg/mL for bioconversions.

### Bioconversion Reactions

The reaction conditions for the bioconversion reactions were derived from Hajnal et al. (2015). The reactions were performed in a biphasic reaction system in 2 mL microreaction tubes. One mg of heat-purified enzyme in 850 μL 50 mM NaPi, pH 7, was supplemented with 150 μL of ethyl acetate containing the substrate to yield a final substrate concentration of 50 mM. The reactions were started by addition of *tert*-butyl hydroperoxide to a final concentration of 150 mM and starting the agitation. The tubes were shaken in a thermomixer at 1,000 rpm and 30°C for 14 h. To stop the reactions, the tubes were cooled on ice and remaining *tert*-butyl hydroperoxide was quenched by addition of 50–100 mg of sodium bisulfite. In the substrate screening experiment (i.e., with the substrates isoeugenol, *trans-*anethole and 2-methyl-1-phenyl-1-propene), no quenching was done as the expected aldehyde reaction products, are known to form insoluble bisulfite adducts with sodium bisulfite. The products were extracted twice with ethyl acetate spiked with 20 mM of *n*-decanol as internal standard (1 μL × 350 μL and 1 μL × 500 μL). The combined organic phases were dried over Na_2_SO_4_ and used for GC and GC–MS analysis. GC analysis was performed using an Agilent 7890B GC system equipped with a flame ionization detector (FID) and a HP-5 19091J-413 column (30 m, 0.32 mm ID, 0.25 μm film, J&W Scientific, Agilent Technologies). The temperature program is given in Supplementary Table 2. Standard curves (5–50 mM) with authentic reference material were used for quantification purpose. The GC–MS analysis was carried out on a 7890A GC System (Agilent Technologies), equipped with 5975C inert XL MSD mass spectrometer (Agilent Technologies) and a HP-5MS column (30 m, 0.25 mm ID, 0.25 μm film, J&W Scientific, Agilent Technologies). For the GC–MS analysis, different temperature programs were used (listed in Supplementary Table 3).

## Results

**FIGURE 1:**
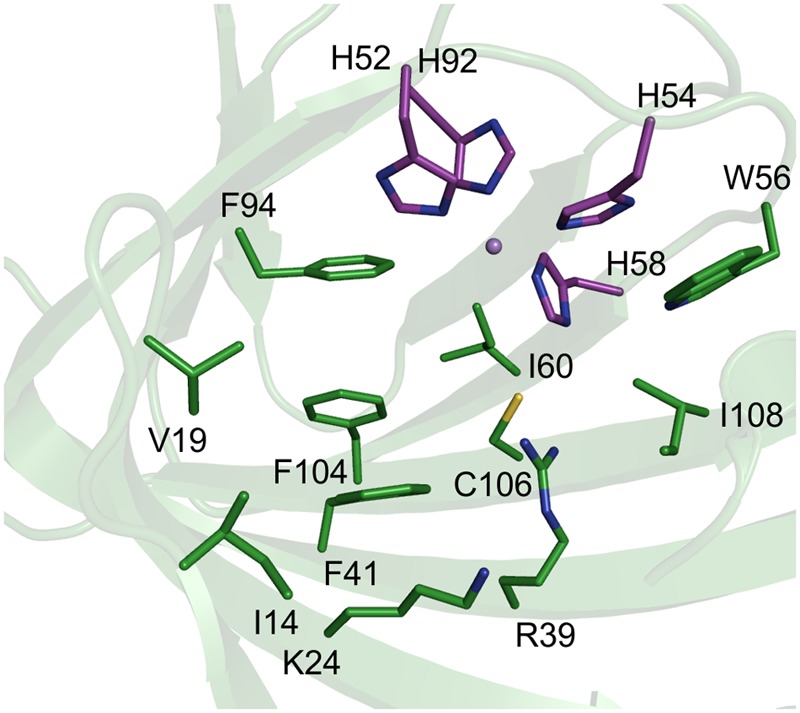
**Active site amino acids depicted as sticks in a cartoon presentation of the structure of TM1459 (pdb-code: 1VJ2, Jaroszewski et al., 2004).** Metal-binding residues are displayed in magenta. The figure was prepared using the program PyMOL.

TM1459 from the thermophilic bacterium *Thermotoga maritima* consists of 114 amino acids and was crystallized as a dimer (Jaroszewski et al., 2004). We previously confirmed the dependency of TM1459 on manganese, which is coordinated by the four histidine residues H52, H54, H58, and H92 located at the base of a large cavity (Hajnal et al., 2015). This cavity is lined by the amino acids Ile14, Val19, Lys24 (all three in the entrance region), Arg39, Phe41, Ile49, Trp56, Ile60, Phe94, Phe104, Cys106 and Ile108, as well as the main chain O of Gly112 and the C-terminal carboxyl group (**Figure 1**). Overall, the amino acids in the binding pocket are mainly hydrophobic and several are bulky. Almost all (nine) amino acids in the active site cavity (excluding the three in the entrance region and the metal binding amino acids) were chosen as targets for site-saturation libraries to increase the activity and broaden the so far quite limited substrate scope of TM1459.

### Assay Development

**FIGURE 2:**
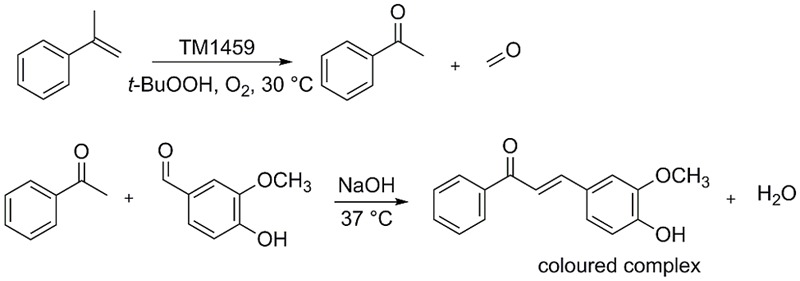
**Biocatalytic oxidative alkene cleavage of α-methylstyrene and formation of aldol condensation product between acetophenone and vanillin**.

**FIGURE 3:**
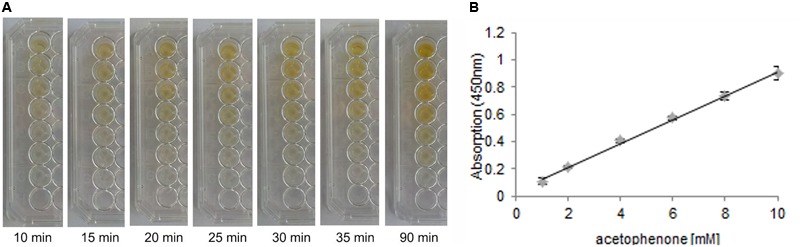
**(A)** Color development of the acetophenone-vanillin reaction with different acetophenone concentrations over time. Concentrations (from top to bottom): 10, 8, 6, 4, 2, 1, 0 mM acetophenone. Reaction conditions to simulate realistic assay conditions: 30 mM *tert*-butyl hydroperoxide, 10–0 mM acetophenone and formaldehyde, 0–10 mM α-methylstyrene, 1 mg/mL *Escherichia coli* BL21 (DE3) Gold lysate with overexpressed CupinX (a cupin protein without alkene cleavage activity) in 50 mM NaPi, pH 7.0, in a total volume of 150 μL. 40 μL of detection solution consisting of 5 M NaOH, 2.5% vanillin and 50% ethanol were added to start the color reaction. **(B)** Standard curve of acetophenone in the range of 1–10 mM (in a total volume of 150 μL in 50 mM NaPi, pH 7.0) detected with 40 μL of detection solution (2 M NaOH, 3.5% vanillin and 10% EtOH) for 30 min at room temperature. Absorption was measured at 450 nm.

To be able to efficiently screen TM1459 libraries for variants with improved activity, a high-throughput assay was developed specifically for the detection of the carbonyl products formed in the cleavage reaction. For the screening, α-methylstyrene was chosen as a model substrate, which is oxidatively cleaved to acetophenone by TM1459 (Hajnal et al., 2015). The detection system is based on the base-catalyzed aldol condensation reaction of vanillin with ketones (Amlathe and Gupta, 1990; **Figure 2**). With the model product acetophenone, a yellow colored complex is formed, while no background reaction occurs with the corresponding alkene substrate. Subsequently, both the alkene cleavage reaction and the detection reaction were adapted for a 96-well plate high-throughput assay and for both reactions the reaction conditions (substrate concentrations, pH, additives, temperature, mixing, data not shown) and reaction times were optimized (**Figure 3A**). In the targeted concentration range of 1–10 mM of acetophenone, the detection assay showed great linearity and reproducibility in the presence of substrates and protein (**Figure 3B**).

In addition, a lysis protocol for the breakage of the cell pellets after deepwell plate fermentation was optimized concerning the volume of lysis solution, lysis time and the addition of varying concentrations of Triton X-100 (0–0.3%, data not shown).

The complete assay consisted of deepwell plate fermentations of variants, followed by disruption of the pelleted cells by a lysis buffer and subsequent centrifugation. The obtained cell-free lysates were transferred to microtiter plates to which in a first step the reaction solution was added and after a certain reaction time, the detection solution was added. No difference in reproducibility or precision was observed whether the color development was measured continuously or after a certain time-point.

**FIGURE 4:**
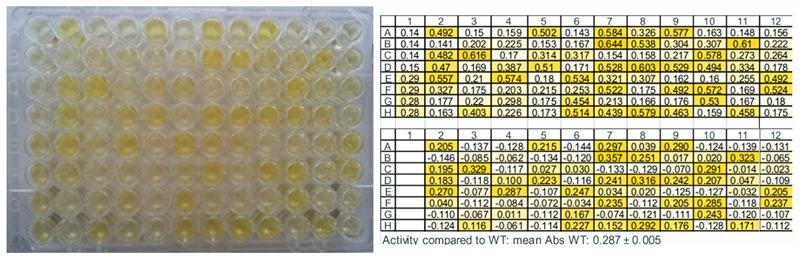
**Example of the microtiter plate assay for the screening of site-saturation libraries.** The first column contains four negative controls (wells 1A–1D), lysate of *E. coli* BL21 (DE3) Gold carrying pET26b(+) vector without insert, and four positive controls (wells 1E–1H), lysate of *E. coli* BL21 (DE3) Gold expressing the wild type enzyme. **(Left)** The picture shows the plate 35 min after addition of the detection solution. **(Right: Top**) Absorbance values of the same plate measured in a plate reader at 442 nm. **(Right: Bottom)** Absorbance difference between WT and variants. The screening was performed with α-methylstyrene in presence of *tert*-butyl hydroperoxide as substrates and vanillin for detection using the standard assay conditions described in Section “Materials and Methods.”

Subsequently, the assay was applied on standard samples, i.e., *E. coli* BL21 (DE3) Gold cells harboring pET26b(+)-TM1459 (positive control) and pET26b(+) without insert (negative control), grown in octuplicate in deepwell plates and lysed. While some background was detected also in the negative control most likely due to carbonyl compounds present in the *E. coli* cells, samples containing active TM1459 were clearly distinguishable with 2–2.5 times higher absorbance (compare first lane in **Figure 4**). The standard deviation of the absorbance of the same sample grown in different wells was usually in the range of below five percent (see also **Figure 4**).

### Screening of Site-Saturation Mutagenesis Libraries

The optimized assay was applied to screen site-saturation libraries of active site amino acids (Arg39, Phe41, Ile49, Trp56, Ile60, Phe94, Phe 104, Cys106, and Ile108) for increased conversion of α-methylstyrene. Differences in color intensity were clearly visible already by eye (**Figure 4**).

**FIGURE 5:**
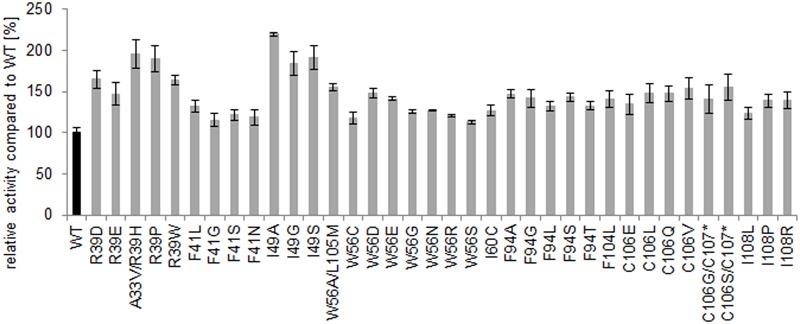
**Microtiter plate assay (re-screening) of the best hits using α-methylstyrene as substrate.** The percentage increase of activity of the variants (mean value of septuplicates on one microtiter plate) refers to comparison with the wild type activity (mean value of septuplicates on the same plate, which is set to 100).

The variants with the highest increase in absorbance compared to the wild type were re-screened in septuplicates and the initial increased activity was confirmed. The variants with the highest gain in absorbance in the re-screening were sequenced. Each library resulted in at least one amino acid exchange with improved activity, especially the positions R39 and I49 resulted in the most active variants with up to 100% increase of activity (**Figure 5**).

In several libraries, variants with the same amino acid exchanges were identified multiple times, whereby the mean absorbance values nicely correlated between hits with the same amino acid exchanges. In some variants also additional mutations generated by random PCR errors were identified. In the C106 library, some of the emerging variants showed a truncation at the C-terminus, which was caused by a nucleotide deletion in the codon at amino acid position 106 that induced a frameshift, which in turn introduced a stop codon at amino acid position 107. The hereby generated variants TM1459-C106S/L107* and TM1459-C106G/L107* (* for stop codon) showed a significantly increased absorbance in the re-screening experiments. Two other variants with additional amino acid exchanges, which were found in library R39 and W56, respectively, are TM1459-A33V/R39H and TM1459-W56A/L105M, respectively, both also showed increased activity in the re-screening. The two positions were deconvoluted and prepared as single variants. No significant increase in activity was observed for TM1459-A33V, but a clear increase of activity was measured for TM1459-L105M (+31% compared to the wild type).

### Combination of Beneficial Amino Acid Exchanges

Based on the results of the library screenings, the identified beneficial amino acid exchanges were combined to variants with two or three amino acid exchanges, as well as C-terminally truncated variants. Moreover, multi-muteins were generated (Supplementary Table 4), thereby combining up to eight single amino acid exchanges.

All variants with two amino acid exchanges except for TM1459-I49A/I108R resulted in increased activity compared to the wild type (data not shown), but only TM1459-I49A/F94A showed a significant increase compared to the underlying single variants [30% (I49A) and 45% (F94A) increase, respectively]. Triple variants did not result in any further increase of activity. Interestingly, TM1459-R39P/F41L/I49A/W56D/F94A/F104L/C106V/I108P showed activity at wild type level, while all other multi-muteins showed no detectable activity at all, most likely due to folding problems as indicated by high amounts of insoluble protein (data not shown). Enzyme variants that were C-terminally truncated at position L107 showed an increased activity compared to the wild type. While the truncated variant without any amino acid exchange is about 20% more active than the wild type, TM1459-C106V, which was C-terminally truncated at position L107, showed the best activity (∼75% increase) among all C-terminally truncated enzyme variants (Supplementary Figure 1).

### Bioconversion Reactions

The most promising TM1459 variants from the screening experiments were produced on larger scale in shaking flasks fermentations and purified by heat treatment (for details about heat treatment see supplemental information and Supplementary Figure 2). In a microtiter plate assay using cell-free lysate and purified protein the improved activity of almost all variants compared to wild type was confirmed. Only the variants R39W and I108R showed decreased activity (Supplementary Figure 3). Moreover, all variants can be purified by heat, however, the loss of activity upon heat treatment varied between 0 and 25% (Supplementary Figure 3).

**FIGURE 6:**
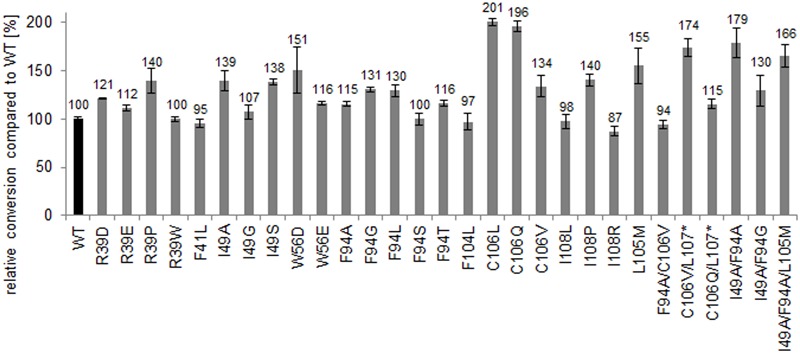
**Relative C = C cleavage activity (conversion %) on α-methylstyrene (based on acetophenone formation) of heat-purified TM1459 and variants thereof (1 mg/mL).** Reaction conditions: 50 mM α-methylstyrene and 150 mM *t*-BuOOH in 15% ethylacetate in 50 mM NaPi, pH 7.0, 30°C and 1,000 rpm for 14 h. 100% refers to conversion of TM1459 (30% conversion).

To quantify the increase in activity of the generated muteins, bioconversion reactions of α-methylstyrene in a biphasic system were conducted with heat-purified proteins. Most variants showed increased conversion compared to the wild type. Especially, the variants TM1459-C106L and -C106Q resulted in significantly improved conversions with a twofold increase compared to the wild type enzyme (**Figure 6**). The increase in conversion using the respective C-terminally truncated variants was less pronounced. Also the previously identified most active double variant TM1459-I49A/F94A led to 79% increased conversion.

**FIGURE 7:**
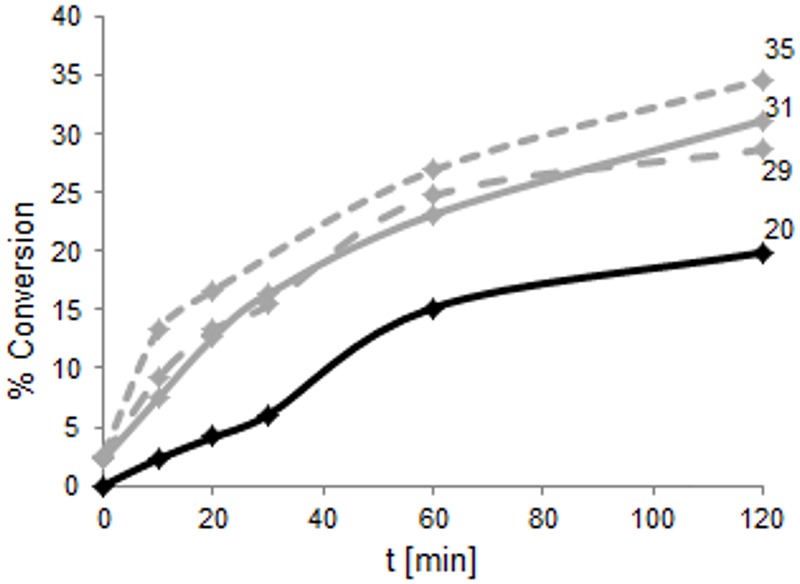
**Time course of the product formation of TM1459 (1 mg total protein) wild type (black) and -C106Q (gray, full line), -I49A/F94A (gray, dashed line), and -C106V/L107^∗^ (gray, dotted line) over the first 2 h of the bioconversion reaction with 50 mM α-methylstyrene and 150 mM *t*-BuOOH in 15% ethylacetate in 50 mM NaPi, pH 7.0, 30°C and 1,000 rpm.** The low product concentration obtained with the wild type in the first 30 min of the reaction accounts for lower accuracy in the quantification, resulting in an apparent non-continuous time profile.

To compare the reaction velocity of the variants in the bioconversion reactions, samples of wild type as well as TM1459-C106Q, -I49A/F94A and -C106V/L107* were analyzed at five points in time (**Figure 7**). It is clearly visible from this data that the new variants were significantly faster than the wild type enzyme, especially in the early phase of the reaction (0–30 min). After 2 h, the variants showed between 29 and 35% conversion, while the wild type showed a conversion of 20%.

**FIGURE 8:**
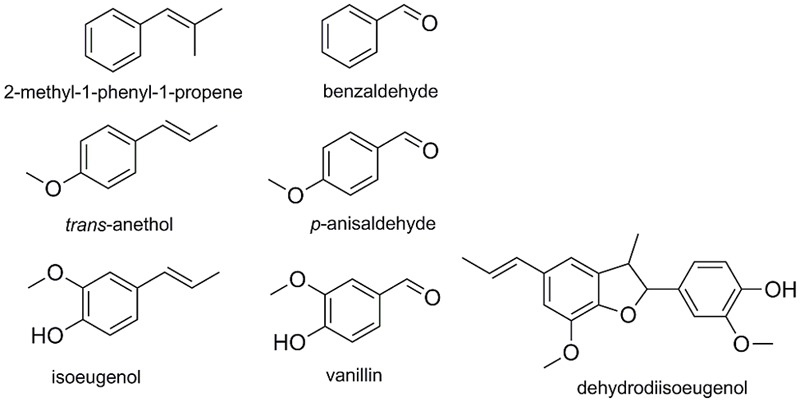
**List of alternative substrates and the respective products**.

To evaluate the effect of the amino acid exchanges on the conversion of additional substrates, compounds similar to α-methylstyrene (and in part already identified as substrates (Hajnal et al., 2015; **Figure 8**) were selected and tested in bioconversion reactions with TM1459-C106Q, -R39P, -I49A/F94A, -C106V/L107* and the wild type enzyme. *p-*Chloro-α-methylstyrene was converted to *p-*Chloroacetophenone with 38% [wild type in accordance to Hajnal et al. (2015)] to 66% (TM1459-C106Q) conversion, clearly confirming the superiority of this variant. 2-Methyl-1-phenyl-1-propene was modestly converted to benzaldehyde with conversions ranging from 2% (WT) to 5% (C106Q). The conversion of *trans-*anethole to *p-*anisaldehyde was 11% with insignificant differences between the different enzyme variants. With *trans-*anethole and 2-methyl-1-phenyl-1-propene, traces of several by-products were observed by GC–MS, with indication of possible oxidation/oxygenation reactions around the C = C double bond. Given the low amount of these side-products, no further characterization was attempted. The conversion of isoeugenol was greatly increased in TM1459-C106V/L107*, but instead of the expected product vanillin, which could only be detected in traces, dehydrodiisoeugenol was produced as main product. With the wild type enzyme, dehydrodiisoeugenol was also detected, but in minute amounts (data not shown).

## Discussion

The strategy adopted to improve activity level of TM1459 in oxidative alkene cleavage reaction by performing site-saturation mutagenesis at selected positions was successfully employed. The results show that especially changes in the C-terminus of TM1459 had a major influence on the activity. Exchanges of the cysteine at position 106 or the isoleucine at position 108 as well as the C-terminal truncation at position 107 are greatly improving enzyme activity (up to 75%). C106 is located close to the metal binding site (side chain SH group 3.14 Å to His58) and interacts with Arg39 (2.91 Å distance), thereby clearly narrowing the cavity. However, an exchange to valine or glutamine does not significantly alter the size of the cavity, or in contrast makes it even smaller. The C-terminus of TM1459 starts with a loop at position 109 that twists the following short α-helical structure toward the protein’s center. While in the available structure, this does not narrow the entrance region or the active site cavity, this loop might be flexible and able to cover or restrict the entrance to the active site. The amino acid exchanges at position 106 and 108 might relocate the terminal α-helical structure and thereby widen the channel to the active site. This hypothesis is supported by the fact that the truncation of the C-terminus at positions 107, which completely removes the terminal helix, induces an increase in activity, which is comparable to the increase in activity caused by amino acid exchanges at positions 106 and 108. In the cases of I49 and F94 the exchange to the smaller amino acids alanine and glycine improved the activity.

Several biotechnology-based ways for the production of vanillin from ferulic acid, eugenol, isoeugenol and even glucose as substrates in various microbial expression hosts have been developed in recent years, some of which are also used on industrial scale (Priefert et al., 2001; Gallage and Møller, 2015). Usually these reactions are catalyzed by multi enzyme systems. Isoeugenol may be transformed by a single enzyme to vanillin upon oxidative cleavage of the double bond adjacent to the aromatic ring. Isoeugenol monooxygenases, e.g., *Iem* from *Pseudomonas nitroreducens* Jin1 (Ryu et al., 2013) and *Iso* from *Pseudomonas putida* IE27 (Yamada et al., 2007) were both shown to catalyze the reaction via epoxidation of the double bond, followed by hydrolysis and cleavage of the resulting diol. Similarly, heme-dependent peroxidases, and importantly the heme prosthetic group alone, can catalyze this reaction in absence of hydrogen peroxide, while diol products were also detected (Mutti et al., 2010). In presence of hydrogen peroxide, lignin peroxidase could only convert *O*-protected isoeugenol to vanillin, while polymerization occurred with the unprotected substrate (ten Have et al., 1998). TM1459 and its variants transformed isoeugenol only in traces to vanillin, and formed dehydrodiisoeugenol as main product, especially the variant TM1459-C106V/L107*. Dehydrodiisoeugenol typically results from oxidative coupling of two molecules of isoeugenol via formation of radical intermediates (Dellagreca et al., 2008; Bortolomeazzi et al., 2010). This type of reaction can be catalyzed enzymatically by peroxidase-H_2_O_2_ systems or laccases (Chioccara et al., 1993; Shiba et al., 2000; Hernández-Vazquez et al., 2011). Interestingly, this oxidative dimerization can also be catalyzed by a combination of H_2_O_2_ and tetraphenylporphyrin manganese(III) chloride, but has not been observed so far in manganese-dependent proteins with a cupin fold. All together it indicates that Mn-containing TM1459 is likely involved in formation of radical species in presence of *t*-BuOOH and oxygen. Mechanistic investigations are ongoing in order to shed light on these two divergent reactions, oxidative C = C bond cleavage and oxidative coupling.

## Conclusion

The successful development of a high-throughput assay for the detection of oxidative alkene cleavage enabled the screening of several site-saturation libraries of active site amino acids. Several variants with increased activity were identified and especially amino exchanges at position 106 resulted in up to twofold increased conversion of α-methylstyrene and *p-*chloro-α-methylstyrene. Thus, we showed that TM1459 performance could be improved by enzyme engineering. In addition, the exceptionally high expression level in *E. coli* and the high temperature stability of the enzyme make TM1459 a promising candidate for industrial application.

## Author Contributions

KS and MH concepted and designed the work; MF and ST performed the experiments; MF, KS, and MH analyzed the data; MF and KS wrote the manuscript; MH and HS critically revised the manuscript.

## Conflict of Interest Statement

The authors declare that the research was conducted in the absence of any commercial or financial relationships that could be construed as a potential conflict of interest.
