# Supplementary material for: Engineering of TM1459 from Thermotoga maritima for Increased Oxidative Alkene Cleavage Activity
Source: Front Microbiol. 2016 Sep 22;7:1511. doi: 10.3389/fmicb.2016.01511 (PMC5031596; doi:10.3389/fmicb.2016.01511)
Supplement: Supplementary file 1 [file Data_Sheet_1.PDF]

frontiers

Supplementary Material

Engineering of TM1459 from *Thermotoga maritima* for increased oxidative alkene cleavage activity

Matthias Fink[1], Sarah Trunk[1], Mélanie Hall[2], Helmut Schwab[3] and Kerstin Steiner[1,*]

*** Correspondence:** Kerstin Steiner: kerstin.steiner@acib.at

1 Supplementary Data

1.1 Protein purification by heat treatment

TM1459 originates from the thermophilic bacterium *Thermotoga maritima,* thus it was expected that TM1459 is a thermostable protein and heat purification might be feasible. Cell-free lysate originating from *E. coli* overexpressing TM1459 (200 µL of lysate with 12 mg/mL protein, 1 vial per time point) was incubated at temperatures between 70 and 95°C for different time periods. The enzyme remained soluble at temperatures up to 80°C and started to precipitate at a temperature of 85°C, while the native *E. coli* proteins precipitated already after a few minutes at 70°C (Figure S2A). In accordance, only samples that showed a lowered enzyme concentration after incubation at temperatures over 80°C, displayed slightly decreased activity (Figure S2B).

2 Supplementary Figures and Tables

2.1 Supplementary Figures

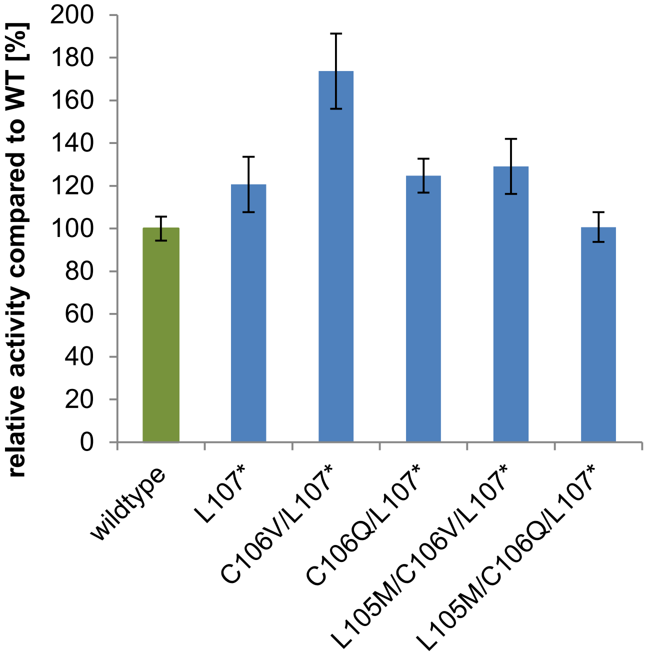

Supplementary Figure 1. Microtiter plate assay of C-terminally truncated variants (in septuplicates) using α-methylstyrene as substrate. Increase of activity compared to wildtype (WT), which is set to 100%. *: mutation to stop-codon. The screening was performed on α-methylstyrene in the presence of *tert*-butyl hydroperoxide and vanillin for detection using the standard assay conditions described in Material and Methods.

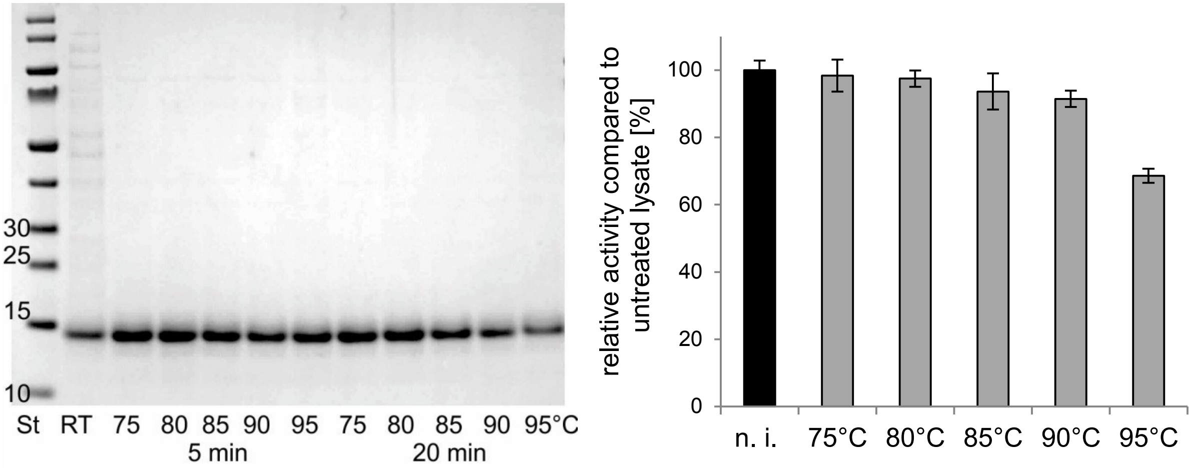

Supplementary Figure 2. Heat purification of TM1459 lysate. A: SDS-PA gel of *E. coli* lysates containing TM1459, which were incubated at different temperatures for 5 minutes and 20 minutes. After incubation the samples were centrifuged for 10 minutes at 20,000x g. Std: PageRuler Prestained Protein Ladder. Lys: Cell-free lysate prior to purification. B: Activity measured by spectrophotometric assay of TM1459 after 20 min of heat purification at temperatures ranging from 75°C to 95°C (grey bars) compared to the untreated sample (black bar, n. i.: not incubated). Note that the protein concentration was not normalized after heat purification, but the same volume was used for each sample. The screening was performed with α-methylstyrene and *tert*-butyl hydroperoxide as substrates and vanillin for detection using the standard assay conditions as described in Material and Methods. The samples were measured in quadruplicates.

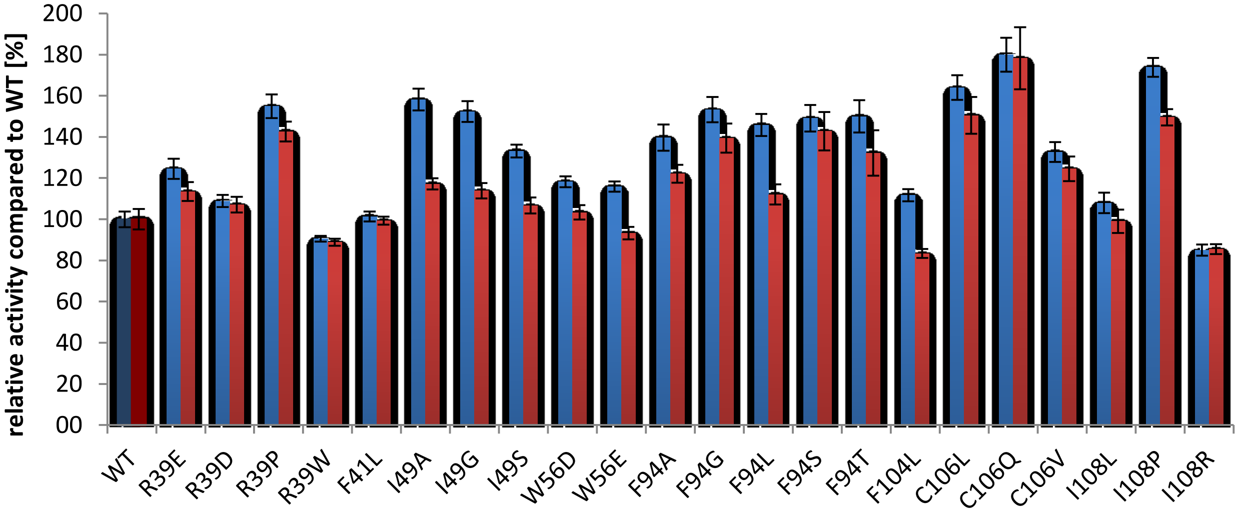

Supplementary Figure 3. Microtiter plate assay of variants with one amino acid exchange before and after heat treatment. The activities are compared to the respective wildtype activity. All samples were expressed in shake flasks, disrupted, centrifuged, (=lysate, blue), heat purified (=purified, red) and the protein concentration diluted to 1 mg/mL after heat purification. The untreated lysates were diluted with the same factor. The screening was performed on α-methylstyrene in the presence of *tert*-butyl hydroperoxide and vanillin for detection using the standard assay conditions described in Material and Methods. Standard deviation of octuplicates.

2.2 Supplementary Tables

Supplementary Table 1. List of primer sequences. Yellow: changed nucleotides. Cupin 2 refers to internal name of TM1459.

Primer name	Primer sequence
synCupin2(Nde)_for	AATCACATATGATTCTGAAACGTGCCTATGATGTTACAC
synCupin2(Hind)_rev	AATCAAAGCTTTTATTCGCCACCTTCTTTCG
Cup2-R39X_for	CCGAATTTTGTGATGNNKCTGTTTACCGTTGAAC
Cup2-R39X_rev	GTTCAACGGTAAACAGMNNCATCACAAAATTCGG
Cup2-F41X_for	GAATTTTGTGATGCGTCTGNNKACCGTTGAACCGGGTG
Cup2-F41X_rev	CACCCGGTTCAACGGTMNNCAGACGCATCACAAAATTC
Cup2-I49X_for	GTTGAACCGGGTGGTCTGNNKGATCGTCATAGCCATC
Cup2-I49X_rev	GATGGCTATGACGATCMNNCAGACCACCCGGTTCAAC
Cup2-W56X_for	GATCGTCATAGCCATCCGNNKGAACATGAAATTTTTG
Cup2-W56X_rev	CAAAAATTTCATGTTCMNNCGGATGGCTATGACGATC
Cup2-I60X_for	CATCCGTGGGAACATGAANNKTTTGTGCTGAAAGGTAAAC
Cup2-I60X_rev	GTTTACCTTTCAGCACAAAMNNTTCATGTTCCCACGGATG
Cup2-F94X_for	CCGAATGAAATTCATGGCNNKCGTAATGATACCGATAG
Cup2-F94X_rev	CTATCGGTATCATTACGMNNGCCATGAATTTCATTCGG
Cup2-F104X_for	CGATAGCGAAGTTGAANNKCTGTGTCTGATTC
Cup2-F104X_rev	GAATCAGACACAGMNNTTCAACTTCGCTATCG
Cup2-C106X_for	GCGAAGTTGAATTTCTGNNKCTGATTCCGAAAGAAGG
Cup2-C106X_rev	CCTTCTTTCGGAATCAGMNNCAGAAATTCAACTTCGC
Cup2-I108X_for	GAATTTCTGTGTCTGNNKCCGAAAGAAGGTGGCG
Cup2-I108X_rev	CGCCACCTTCTTTCGGMNNCAGACACAGAAATTC
Cup2-I49A_for	GTTGAACCGGGTGGTCTGGCTGATCGTCATAGCCATC
Cup2-I49A_rev	GATGGCTATGACGATCAGCCAGACCACCCGGTTCAAC
Cup2-F94A_for	GTTGAACCGGGTGGTCTGGCTGATCGTCATAGCCATC
Cup2-F94A_rev	GATGGCTATGACGATCAGCCAGACCACCCGGTTCAAC
Cup2-A33V_for	CTGATTGGTCTGAAAGATGTTCCGAATTTTGTGATGCGTC
Cup2-A33V_rev	GACGCATCACAAAATTCGGAACATCTTTCAGACCAATCAG
Cup2-L107M_for	CGAAGTTGAATTTCTGTGTATGATTCCGAAAGAAGGTGG
Cup2-L107M_rev	CCACCTTCTTTCGGAATCATACACAGAAATTCAACTTCG
Cup2-L105M_for	CCGATAGCGAAGTTGAATTTATGTGTCTGATTCCGAAAGAAG
Cup2-L105M_rev	CTTCTTTCGGAATCAGACACATAAATTCAACTTCGCTATCGG
Cup2-L107*_rev	GCCGCAAGCTTTTAACACAGAAATTCAACTTCGCTATCGG
Cup2-C106V/L107*_rev	GCCGCAAGCTTTTAAACCAGAAATTCAACTTCGCTATCGG
Cup2-C106Q/L107*_rev	GCCGCAAGCTTTTACTGCAGAAATTCAACTTCGCTATCGG
Cup2-L105M/C106Q/L107*_rev	GCCGCAAGCTTTTACTGCATAAATTCAACTTCGCTATCGG
Cup2-L105M/C106V/L107*_rev	GCCGCAAGCTTTTAAACCATAAATTCAACTTCGCTATCGG

Supplementary Table 2. Temperature program used for GC analysis

	Rate (°C/min)	Value (°C)	Hold Time (min)	Run Time (min)
**Initial**		80	0.5	0.5
**Ramp 1**	10	160	0	8.5
**Ramp 2**	20	280	2	16.5

Supplementary Table 3. Temperature programs used in the GC-MS analysis. All samples were first measured with program 1 and additionally with other programs if necessary (as indicated).

		Rate (°C/min)	Value (°C)	Hold Time (min)	Run Time (min)
**Program 1 (all samples)**	Initial		100	0.5	0.5
	Ramp 1	10	300	0	20.5
**Program 2 (isoeugenol)**	Initial		200	0.5	0.5
	Ramp 1	5	300	2	22.5
**Program 3 (2-methyl-1-phenyl-1-propene, *t*-anethole)**	Initial		80	1	1
	Ramp 1	20	300	1	13
**Program 4 (indole)**	Initial		100	0.5	0.5
	Ramp 1	15	300	1	15.833

Supplementary Table 4. List of all variants generated in course of the third mutagenesis round. Asterisks represent mutations to a stop-codon.

**Two amino acid exchanges**	**C-terminal truncated variants**
R39P/I49A	L107*
R39P/F94A	C106Q/L107*
I49A/F94A	C106V/L107*
I49A/F94G	L105M/C106Q/L107*
I49A/W56D	L105M/C106V/L107*
I49A/F104L	**Multiple amino acid exchanges (“multi-muteins”)**
I49A/C106V	R39P/F41L/I49A/W56D/F94A/F104L/C106V/I108P
I49A/I108R	R39P/I49A/W56D/F94A/C106V/I108R
W56D/F94A	R39P/I49A/F94A/C106V
F94A/F104L	I49A/F94A/C106V/L107*
F94A/C106V	I49A/F94A/L105M/C106Q/L107*
F94A/I108R	I49A/F94A/L105M/C106V/L107*
**Three amino acid exchanges**	I49A/F94A/C106Q/L107*
I49A/F94A/L105M	
I49A/F94A/I108P	
